# Long-term *in vitro* culture of grape berries and its application to assess the effects of sugar supply on anthocyanin accumulation

**DOI:** 10.1093/jxb/ert489

**Published:** 2014-01-29

**Authors:** Zhan Wu Dai, Messaoud Meddar, Christel Renaud, Isabelle Merlin, Ghislaine Hilbert, Serge Delrot, Eric Gomès

**Affiliations:** ^1^INRA, ISVV, UMR 1287 EGFV, 33882 Villenave d’Ornon, France; ^2^Université de Bordeaux, ISVV, UMR 1287 EGFV, 33882 Villenave d’Ornon, France

**Keywords:** Anthocyanins, grape quality, *in vitro*, secondary metabolism, sugar signalling, *Vitis*.

## Abstract

Grape berry development and ripening are under complex regulation by the nutrients, hormones, and environment cues sensed by the berry. However, the biochemical and molecular mechanisms underlying these types of regulation are poorly understood. A simplified but realistic model system that enables fruit growth conditions to be modulated easily will facilitate the deciphering of these mechanisms. Here, an *in vitro* culture system of intact detached grape berries was developed by coupling the production of greenhouse fruiting-cuttings and *in vitro* organ culture techniques. ^13^C and ^15^N labelling experiments showed that this system enables the intact detached berries actively to absorb and utilize carbon and nitrogen from the culture medium. It was further used to study the effects of sugars on anthocyanin accumulation. A sucrose concentration >2% could induce anthocyanin synthesis in the absence of additional exogenous abscisic acid. The higher the sucrose concentration, the earlier was the induction of anthocyanin accumulation. Glucose, fructose, and sucrose increased anthocyanin accumulation, with glucose and fructose being more effective than sucrose. This increase was not due to an increase in its precursor level, since the phenylalanine content was decreased by a high sugar supply. Instead, genome-wide transcriptome analysis suggests that the sugar-induced enhancement of anthocyanin accumulation results from altered expression of regulatory and structural genes (especially UDP-glucose:anthocyanidin 3-*O*-glucosyltransferase), together with massive reprogramming in signalling transduction pathways. This *in vitro* system may serve to study the response of berry composition to nutrient factors and hormones, and their interaction with environmental factors (e.g. light and temperature), which can all be finely tuned and controlled.

## Summary

A long-term *in vitro* culture system of intact grape berries was developed which can serve to study the response of berry composition to various trophic factors, shown by sugar regulation of anthocyanin accumulation.

## Introduction

Grape berry development and metabolism are under complex regulation by nutrients and hormones, and by the environmental cues sensed by the berry (Geros *et al.*, 2012). The study of the effects of these factors on berries still attached to intact plants is very challenging because of the large size of the plants, because of interplant, intercluster, and interberry variability, and because the fine control of the nutrient and hormonal import, and the environment is complicated. An experimental system allowing the dissection of the parameters that control fruit ripening is desirable.

Sugars and anthocyanins are two classes of compounds playing key roles in wine quality. Grape sugars and fermentation techniques determine the alcohol level of the wine, while anthocyanins (i.e. colour pigments) of red wine grape cultivars directly impact wine colour. Sugars regulate anthocyanin accumulation in grape berry (reviewed by Agasse *et al.*, 2009). Sucrose enhances anthocyanin synthesis in intact detached berries (Pirie and Mullins, 1976; Roubelakis-Angelakis and Kliewer, 1986) and in grape cell suspensions (Yamakawa *et al.*, 1983; Cormier *et al.*, 1990; Larronde *et al.*, 1998). In grape berry slices, high sugar levels induce anthocyanin accumulation within 96h (Zheng *et al.*, 2009). However, high sucrose concentrations tend to inhibit anthocyanin accumulation in halved Olympia (*Vitis labruscana*) berries cultured *in vitro* (Hiratsuka *et al.*, 2001). A recent study reported that anthocyanins were only synthesized in berries cultured in media with high (10% compared with 2%) sucrose supplemented with 10–200 μM abscisic acid (ABA), but not in media solely containing high sucrose (Gambetta *et al.*, 2010). These authors emphasized the essential role of the simultaneous presence of sucrose and ABA for the induction of anthocyanin accumulation. Indeed, in field-sampled berries, there is a tight positive correlation between sugar and anthocyanin content (González-SanJosé and Diez, 1992; Castellarin *et al.*, 2011).

The different anatomical complexity of these systems may explain the conflicting results regarding the relationship between sugar and anthocyanin contents. In cell suspensions, the sites (i.e. the cells) of sugar and anthocyanin accumulation are the same, while this is not the case in intact grape berries. In an intact berry, anthocyanins accumulate in the vacuoles of skin cells (Gomez *et al.*, 2009; Francisco *et al.*, 2013), while sugars are mainly accumulated in the flesh cells. These compartmental differences may lead to different patterns of sugar regulation of anthocyanin synthesis in cell suspensions, sliced berries, halved berries, intact detached berries, and field-sampled berries. As a result, care should be taken when extrapolating results from cell suspensions to intact grape berries. *In vitro* cultured grape berries may be a suitable model system, since they provide an intermediate situation between cell suspensions and berries attached to the parent plants. This experimental model represents the berry compartmentation between flesh and skin well, and it is simpler than whole plants where the manipulation of carbon supply may be complicated by the wood reserves (Candolfi-Vasconcelos *et al.*, 1994). It also allows a fine-tuned control of abiotic environmental factors (light, temperature, etc.). In this context, the use of *in vitro* cultured intact berries for studying the effect of sugars on anthocyanin accumulation could provide more realistic information than cell suspensions for viticultural applications.

The mechanisms underlying the sugar regulation of anthocyanin synthesis remain largely unknown. A few studies demonstrated that this regulation is mediated by the expression of genes involved in anthocyanin synthesis (Solfanelli *et al.*, 2006; Loreti *et al.*, 2008; Zheng *et al.*, 2009). In an *Arabidopsis* mutant impaired in phloem unloading and thereby accumulating high sugar levels, anthocyanins accumulate in the leaves as a result of sucrose-induced expression of anthocyanin biosynthesis-related genes (Solfanelli *et al.*, 2006). In sliced grape berries, sucrose up-regulates *F3H* (*flavonone 3-hydroxylase*) expression, coinciding with enhanced anthocyanin levels (Zheng *et al.*, 2009). This enhancement may be mediated by Ca^2+^-calmodulin and hexokinase (Vitrac *et al.*, 2000). Whether high sugar also modifies the composition of anthocyanins in addition to increasing their amount is still unknown. A simple but realistic model system that enables easy modulation of the sugar status in grape berry is therefore desirable to decipher the biochemical and molecular mechanisms of this regulation.

Intact grape berries cultivated *in vitro* have been used to study the synthesis of monoterpene glycoside (Bravdo *et al.*, 1990), the growth response to carbon source and sucrose concentration (Pérez *et al.*, 2000), the effects of sugar (Pirie and Mullins, 1976; Roubelakis-Angelakis and Kliewer, 1986), temperature and light (Azuma *et al.*, 2012), and ABA (Deis *et al.*, 2011) on anthocyanin biosynthesis. However, reports using *in vitro* cultured berries to study anthocyanin synthesis are rare and are limited to short-term experiments (~10 d), probably due to contamination problems in long-term experiments (Hiratsuka *et al.*, 2001). One of the few studies which have been conducted reported a 23 d *in vitro* culture of intact detached berries (Gambetta *et al.*, 2010). However, these studies did not quantify the exchanges between the berries and the culture medium, especially the uptake of sugars, and did not cover the full-length ripening period (50–60 d, on average, for field-grown berries). Therefore, it is worth developing and characterizing an *in vitro* berry culture system that enables long-term culture covering the whole ripening processes. The present study describes the development of a two-step *in vitro* culture system, which couples the use of fruiting-cuttings with organ *in vitro* culture. This system was characterized by ^13^C and ^15^N tracing, and its usefulness for berry ripening biochemical and molecular regulation was demonstrated by an integrative study detailing the effects of sugar supply on anthocyanin accumulation.

## Materials and methods

### Source of explants

Explants of grape berries were obtained from either field-grown grapevines or greenhouse-grown fruiting-cuttings of *Vitis vinifera* L. cv. Cabernet Sauvignon. The field-grown grapevines were 21 years old, spur pruned, with a density of 1.6 m between rows and 1 m between plants. Culture management followed local standards, with phytochemical treatments about twice a month from the end of April to the middle of August and thereafter no treatment. The fruiting-cuttings (i.e. only one primary shoot axis with a single cluster per plant) were prepared as described in Mullins and Rajasekaran (1981) and grown in a naturally illuminated and semi-regulated greenhouse (mean seasonal temperature amplitude 20–35 °C) with chemical treatments every 2 weeks.

### Set-up of the *in vitro* berry culture system

Different sterilization durations in 70% ethanol and NaClO (1, 2, or 3% available chlorine) were tested for berries sampled from greenhouse-grown fruiting-cuttings or field-grown vines during pre-veraison and post-veraison stages using solid or liquid media.

For all sterilization trials, the nutritive medium used (M 0221, Duchefa) was composed of macro- and microelements (Murashige and Skoog, 1962) supplemented with 2% sucrose, 0.025% N-Z-Amine A (C7290, Sigma), and vitamins (myo-inositol 100mg l^–1^, nicotinic acid 1mg l^–1^, pantothenic acid 1mg l^–1^, biotin 0.01mg l^–1^, pyridoxine HCl 1mg l^–1^, and thiamine HCl 1mg l^–1^). The pH was adjusted to 5.8 with 0.5M NaOH. For solid medium, 0.9% agar (HP 696, Kalys) was also added to the medium before autoclaving (120 °C, 20min). After autoclaving, 4ml of solid medium was distributed in 6-well plates (353046, Dutscher), while 150ml of liquid medium was distributed into a tissue culture box equipped with special filters allowing gas exchange (E 1650, Duchefa). To make sure that berries were kept in close contact with the liquid medium, home-made floaters with tip plates and polystyrene were sterilized with 10% NaClO and 90% ethanol during 20min and then installed in the tissue culture box (Supplementary Fig. S1 available at *JXB* online).

Berries with a pedicel ~3mm long were first excised from grape clusters and put into ethanol and NaClO solutions for different durations (Table 1). After rinsing with deionized water, the berries were dipped in a 20mM EDTA solution to prevent plugging of sieve tubes by callose synthase, a strictly calcium-dependent enzyme, and re-cut at the pedicel to ~2mm in the EDTA solution. Berries were then quickly put into solid or liquid media, and transferred into a culture chamber with constant temperature of 26±0.5 °C, a light period of 16h/8h day/night, and light of ~50 μmol m^–2^ s^–1^. Each treatment included 24 berries, and contamination was checked every day until all berries were contaminated or no new contamination occurred.

**Table 1.** Optimization of sterilization for the system of in vitro culture for grape berries

Explant source	Year	Experiment number	Explant age^*a*^	Explant colour	Sterilization	Culture duration (d)	Contamination rate
Greenhouse	2010	2010.1	20	Green	Ethanol 70% 1 min	20	0%
				Green	Ethanol 70% 2 min	20	0%
				Green	Ethanol 70% 3 min	20	0%
				Green	Ethanol 70% 2 s+NaClO 1% 1 min	20	0%
				Green	Ethanol 70% 2 s+NaClO 1% 2 min	20	0%
				Green	Ethanol 70% 2 s+NaClO 1% 3 min	20	0%
				Green	Ethanol 70% 2 s+NaClO 2% 1 min	20	0%
				Green	Ethanol 70% 2 s+NaClO 2% 2 min	20	0%
				Green	Ethanol 70% 2 s+NaClO 2% 3 min	20	0%
		2010.2	50	Green	Ethanol 70% 2 s+NaClO 1% 1 min	45	5.6%
		2010.3	87	Red	Ethanol 70% 2 s+NaClO 1% 1 min	30	14.3%
	2011	2011.1	44	Green	Ethanol 70% 2 s+NaClO 1% 2 min	90	1.67%
		2011.2	45	Green	Ethanol 70% 2 s+NaClO 1% 2 min	60	0
Vineyard	2010	2010.4	32	Green	Ethanol 70% 2 s+NaClO 1% 1 min	3	100%
		2010.5	35	Green	Ethanol 70% 10 s+NaClO 1% 2 min	7	100%
				Green	Ethanol 70% 10 s+NaClO 1% 4 min	7	100%
				Green	NaClO 1% 10 s+ethanol 70% 2 min	7	100%
				Green	NaClO 1% 10 s+ethanol 70% 4 min	7	100%
		2010.6	42	Green	Ethanol 70% 10 s+NaClO 1% 6 min	7	100%
				Green	Ethanol 70% 10 s+NaClO 1% 8 min	7	100%
				Green	Ethanol 70% 10 s+NaClO 1% 10 min	7	100%
		2010.7	50	Green	Ethanol 70% 10 s+NaClO 2% 5 min	7	100%
				Green	Ethanol 70% 10 s+NaClO 2% 10 min	7	100%
				Green	Ethanol 70% 10 s+NaClO 2% 15 min	7	100%
				Green	Ethanol 70% 10 s+NaClO 2% 20 min	7	100%
		2010.8	56	Veraison	Ethanol 70% 10 s+NaClO 3% 15 min	42	67%
		2010.9	59	Veraison	Ethanol 70% 10 s+NaClO 3% 15 min	37	65%

^*a*^ Explant age in days after flowering.

### Characterizing nutrient uptake and utilization by ^13^C and ^15^N labelling

Grape berries (cv. Cabernet Sauvignon) from greenhouse-grown fruiting-cuttings were sampled when the first berry in a cluster changed its colour (start of veraison). After excision from the cluster, berries were sterilized as described above and put into *in vitro* culture in liquid media containing ^13^C- and ^15^N-labelled substrates. Two different labelling liquid media were used: M1 with 5% (w/w) glucose-^13^C1 (99% atom ^13^C)+95% (w/w) glucose with a natural carbon isotope abundance and 100% (w/w) ^15^NH_4_^15^NO_3_ (10% atom ^15^N); and M2 with 1% (w/w) sucrose-^13^C12 (99% atom ^13^C)+99% (w/w) sucrose with a natural carbon isotope abundance and 100% (w/w) ^15^NH_4_^15^NO_3_ (10% atom ^15^N). The final media contained 174mM glucose (M1) or sucrose (M2) plus 30mM NH_4_NO_3_.

About 7ml of medium was poured into sterile plastic vials (height 6.8cm, diameter 3.3cm), and single berries were maintained floating on the medium with a home-made polystyrene floater (Supplementary Fig. S2 available at *JXB* online). In total, 48 berries were labelled, with 24 berries for each medium.

The berries were sampled after 0, 1, 5, 15, and 30 d of *in vitro* culture. First, they were rinsed three times with deionized water in order to exclude any isotopic contamination from the medium. Afterwards, each berry was carefully separated into pedicel, seed, and pericarp (including the skin and pulp), and the seeds were further rinsed three times with deionized water to avoid any contamination from the pulp. All samples were then stored at –80 °C for later analysis. For each sampling date and condition, three replicates of two berries were collected. Meanwhile, the media of all treatments were also sampled.

Frozen berries were ground in liquid nitrogen, freeze-dried, and weighed. About 0.15–1.5mg of fine powder was used to analyse nitrogen and carbon content, and ^15^N and ^13^C abundances. These parameters in the media were determined with 10 μl of media after drying in an oven over 72h. Total nitrogen and carbon as well as the abundance of ^15^N and ^13^C were analysed with an elemental analyser EA3000 (EuroVector, Germany) coupled to an isotopic ratio mass spectrometer IsoPrime (Elementar, Germany). The machine was calibrated with international isotopic standards (IAEA, Vienne, Australia) of V-PDB for carbon and atmospheric N_2_ for nitrogen. In addition, free amino acids were measured using an HPLC system after derivatization with 6-aminoquinolyl-*N*-hydroxysuccinimidyl carbamate as described in Cohen and Michaud (1993) and Pereira *et al.* (2006).

Uptake and distribution of newly incorporated ^13^C and ^15^N in a berry was calculated as follows for carbon.

Absolute molar proportions (*A*_c_) of the heavy isotope per 100 atoms (also named abundance) is defined as:

$$A_{c}\text{\%}=\frac{{}^{13}C}{{}^{13}C+\;{\;}^{12}C}\times 100$$

The relative specific allocation (RSA) was then calculated using *A*_c_%:

$$RSA_{C}\text{\%}=\frac{A_{C\_labelled}-A_{C\_labelled}}{A_{C\_labelled}-A_{C\_labelled}}\times 100$$

where the subscript stands for the *A*_c_% of labelled samples (labelled), of unlabelled samples (field-sampled berries) with natural isotope abundance (unlabelled), and of the culture medium (medium). Finally, the quantity of newly incorporated carbon for each organ was calculated with its dry mass (DW) and total carbon content (C%):

$$New\;C\;content\left(mg\;organ^{-1}\right)=RSA_{C\_organ}\text{\%}\times DW_{organ}\times C{\text{\%}}_{organ}$$

The same calculation was made for nitrogen by replacing carbon with nitrogen in the above equations.

### Effect of sugar concentration on anthocyanin accumulation (experiment 1)

Grape berries (cv. Cabernet Sauvignon) from greenhouse-grown fruiting-cuttings were collected at 40 d after flowering (green and hard berry) and cultured *in vitro* with solid MS medium (Murashige and Skoog, 1962) and five concentrations of sucrose (2, 4, 8, 12, and 16%). Sterilization was performed as described in the above section. Berries were sampled after 60 d and 90 d of *in vitro* culture. Each treatment included three replicates and each replicate consisted of 6–8 berries.

On both harvest dates, berries were removed from the medium, rinsed with deionized water, weighed, and stored at –80 °C for further analysis. Before chemical analysis, berries (without seed and pedicel) were ground in liquid nitrogen using a mortar and pestle. Sugars (fructose and glucose), free amino acids, and anthocyanins were analysed by using fresh or freeze-dried sample powders. Sugar levels were measured enzymatically with an automated micro-plate reader (ELx800UV, BioTek Instruments Inc., France), as described by Gomez *et al.* (2007). Free amino acids were measured as described in the ^13^C and ^15^N labelling experiment. Anthocyanins were determined using an HPLC system after extraction with methanol and 0.1% (v/v) HCl as described in Hilbert *et al.* (2003) and Acevedo De la Cruz *et al.* (2012), with malvidin-3-glucoside (Oenin, HPLC grade, Extrasynthese, Genay, France) as standard. Briefly, each single anthocyanin was detected with a Summit HPLC System equipped with a P680 pump, an ASI-100T™ auto-sampler, and a UVD 340U UV-Vis detector at 520nm (Dionex Corporation, Sunnyvale, CA, USA). Separation was achieved after injecting a 20 μl extraction to a reverse-phase Ultrasphere ODS column 25 cm×4.6mm, 5 μm particle size with an Ultrasphere ODS guard column 4.5 cm×4.6mm (Beckman Instruments Inc., Fullerton, CA, USA), at 25 °C. A binary gradient elution (70min) with a 0.6ml min^–1^ flow rate was used, starting with 75% eluant A [10% formic acid in water (v/v)] and ending with 90% eluant B [10% formic acid and 30% acetonitrile in water (v/v)]. The Chromeleon software (version 6.60, Dionex Corporation) was used to calculate the peak area. Identification and peak assignment of each single anthocyanin were based on comparison of their retention times and UV-Vis spectrometric data with those of pure standards.

### Effect of sugar forms (glucose, fructose, and sucrose) on anthocyanin accumulation (experiment 2)

Grape berries (cv. Cabernet Sauvignon) from greenhouse-grown fruiting-cuttings were harvested 55 d after flowering (green berry, 11 d before véraison) and cultured *in vitro* in liquid MS medium with three concentrations (58, 234, and 468mM) of three different sugar forms (glucose, fructose, and sucrose). The concentrations were chosen based on the results of experiment 1 and corresponded to 2, 8, and 16% sucrose. To exclude any effect of osmotic potential, the concentrations of glucose, fructose, and sucrose were expressed on a molar basis, and non-assimilable mannitol was used to reach the same osmotic potential for all media (Supplementary Table S2 available at *JXB* online). Other culture manipulations were as described in experiment 1. Berries were sampled after 48 d of *in vitro* culture. Each treatment had three replicates and each replicate had six berries. The numbers of berries that showed visible colour change were counted. Sampling and chemical analysis were as described in experiment 1. In addition, these berries were also used for transcriptmic studies.

### Effect of sugar supply on transcriptome reprogramming for berries cultured *in vitro*

Microarray analysis was conducted to analyse transcriptome changes in berries cultured in high (468mM) and low (58mM) glucose media. Total RNA of three biological replicates per treatment were extracted from 1g of fresh fine powder as described in Reid *et al.* (2006). Total RNA was then treated with DNase I (RQ1 RNase-Free DNase, Promega, Madison, USA), acid phenol:chloroform:isoamyl alcohol mix (25:24:1), and a Nucleospin RNA Clean-up XS Kit (Macherey-Nagel, Germany) to remove genomic DNA. After quality control, 5 μg of RNA was reverse transcribed to synthesize cDNA (SuperScript One-Cycle cDNA kit, Invitrogen, Carlsad, USA). Finally, 1 μg of cDNA was labelled and hybridized to the NimbleGen 12×135K whole grape genome microarray 090918_Vitus_exp_HX12 (NimbleGen, Madison, WI, USA) following the manufacturer’s instructions.

Microarray data were analysed using R (R Development Core Team, 2010) and Bioconductor tools (Gentleman *et al.*, 2004). After quality control with the ArrayQualityMetrics package (Kauffmann *et al.*, 2009), expression intensities were normalized with RMA function with the oligo package (Carvalho and Irizarry, 2010). Genes differentially expressed between high and low glucose concentration in the culture media were identified using the moderate *t*-test in the LIMMA package (Smyth, 2005). Genes with a >2-fold up- or down-regulation and an adjusted *P*-value <0.05 after Benjamini and Hochberg (1995) false discovery rate correction were considered significantly differentially expressed between the two treatments. Mapman software (Thimm *et al.*, 2004) was used to visualize gene expression data in the context of their corresponding metabolic pathways. Significant enrichments of functional categories of the MapMan ontology in the significantly differentially expressed genes were tested by using Mefisto Version 0.23beta (http://www.usadellab.org) with a Bonferroni correction for multiple tests. All the data have been submitted in MIAME-compliant form within the ArrayExpress database (http://www.ebi.ac.uk/arrayexpress/; accession no. E-MTAB-1634).

## Results and Discussion

### Design of a long-term *in vitro* culture system for grape berries

Berries from fruiting-cuttings grown in a greenhouse could be readily sterilized with 70% ethanol and 1% NaClO under all tested combinations of rinsing durations (Table 1). The contamination rate was quite low for green berries (0–5.6%) and slightly higher for red berries (14.3%). Based on these observations, a sterilization procedure of 70% ethanol (2 s)+1% NaClO (2min) was chosen for the following experiments in 2011. The results showed that this sterilization was effective, with only 1.67% contamination during 90 d of culture *in vitro*. In contrast, berries from field-grown vines were very difficult to sterilize, with 100% contamination observed within 7 d for green berries sterilized with 70% ethanol (10 s)+2% NaClO (20min) (Table 1). Even when sterilized with 70% ethanol for 10 s+3% NaClO for 15min, coloured berries still reached 67% contamination after 42 d of culture *in vitro*. The difficult sterilization observed for grape berries from field-grown vines is in agreement with the data of Hiratsuka *et al.* (2001), who sterilized field-grown berries with 1% NaClO for 10min and observed fungal infection within 5 d. On the other hand, the performance of berries from the fruiting-cuttings is most probably related to the fact that they are more frequently treated with fungicide and pesticide and grown in a relatively closed space, and thereby are likely to contain fewer fungal mycelia and spores compared with field-harvested fruits. Therefore, only berries from fruiting-cuttings grown in the greenhouse were used for further experiments.

### Detached berries can actively take up and metabolize carbon and nitrogen in the *in vitro* culture system

**Fig. 1.**
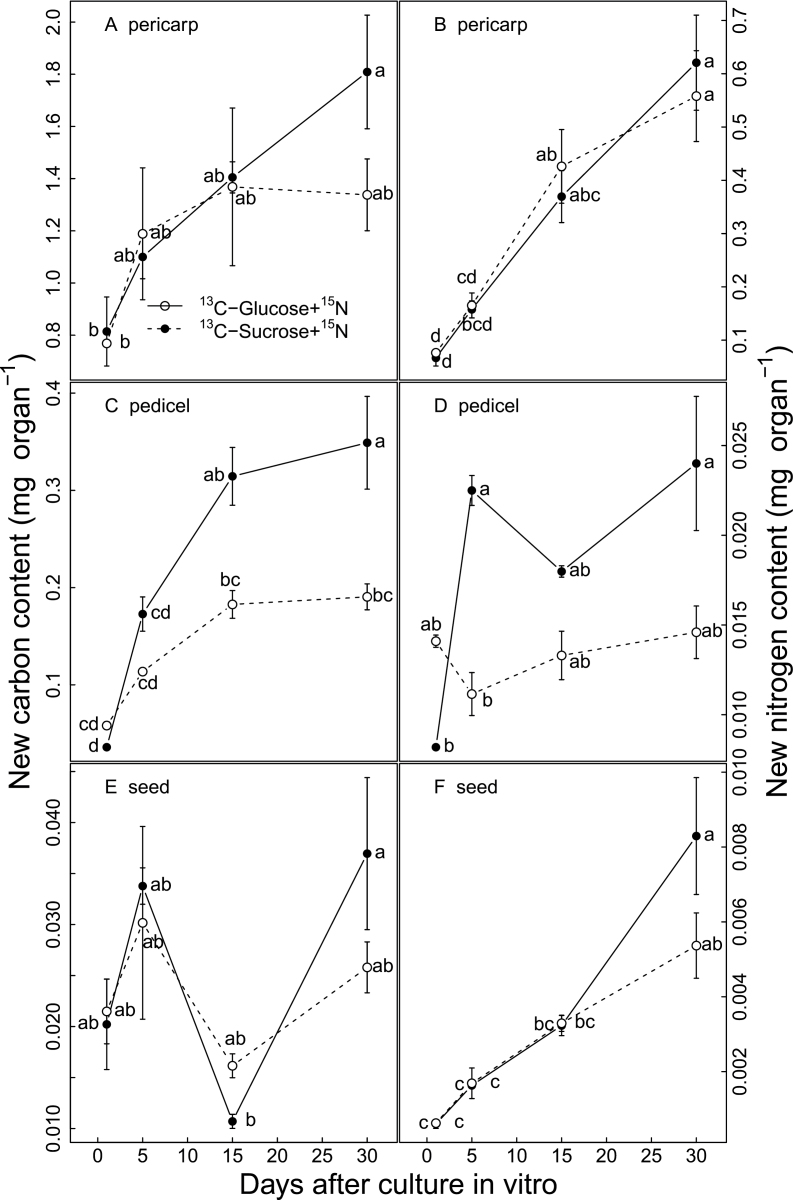
Carbon (A, C, E) and nitrogen (B, D, F) imported into pericarp (A, B), pedicel (C, D), and seed (E, F) from the culture medium as a function of *in vitro* culture duration. Each value corresponds to a mean ±standard error (*n*=3). Different letters indicate significant differences among comparisons of date and medium at *P*<0.05.

It was checked whether the berries cultured *in vitro* could actively absorb nutrients (sugars and nitrogen) from the medium. To this end, ^13^C-labelled glucose and sucrose and ^15^N-labelled NH_4_NO_3_ were used to trace the transport of carbon and nitrogen from the culture medium to the detached berries (Fig. 1). Over the tracing period, carbon and nitrogen concentrations in each medium remained relatively constant (data not shown). Both carbon and nitrogen were absorbed from the culture medium by the detached berries. About 0.8mg of carbon and 0.07mg of nitrogen were imported into berry pericarp after 1 d of *in vitro* culture, and these values gradually increased ~2-fold for carbon (to 1.6mg) and ~8-fold for nitrogen (to 0.59mg) after 30 d culture (Fig. 1A, B). Carbon was also incorporated into the pedicel, increasing from 0.04mg to 0.25mg throughout 30 d culture *in vitro* (Fig. 1C), whereas the newly incorporated nitrogen in the pedicel remained relatively stable at 0.02mg (Fig. 1D). Seed obtained lower amounts of the newly imported carbon (~0.02mg) and nitrogen (~0.003mg) (Fig. 1E, F). The sugar type (glucose or sucrose) in the medium had a minor effect on carbon uptake and did not affect nitrogen uptake (Fig. 1).

**Fig. 2.**
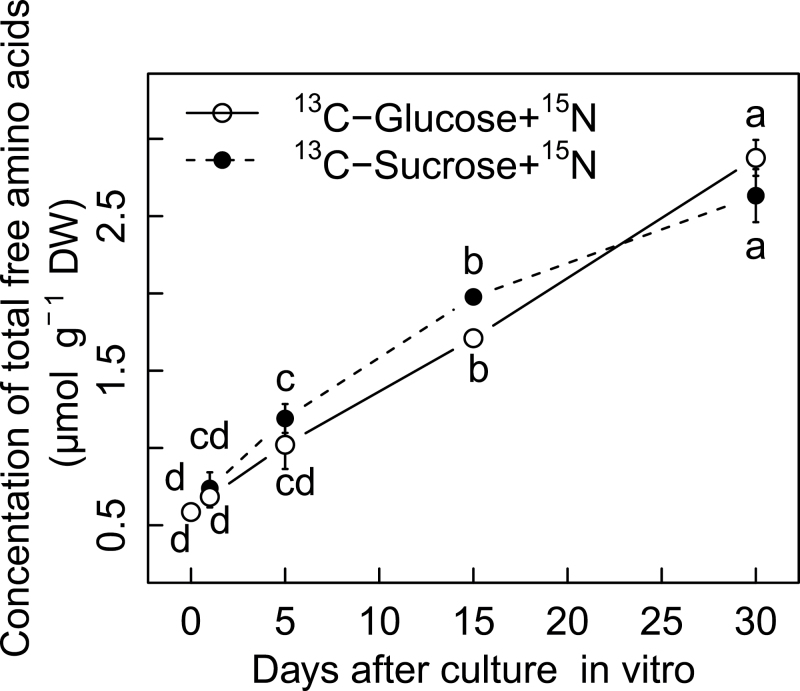
Concentration of total free amino acids in grape berries cultured *in vitro* as a function of culture duration. Each value corresponds to a mean ±standard error (*n*=3). Letters indicate significant differences among comparisons of date and medium at *P*<0.05.

In parallel with significant nitrogen absorption into the berries, the concentration of total free amino acids increased dramatically from 0.56 μmol g^–1^ DW to 2.75 μmol g^–1^ DW after 30 d culture (Fig. 2). Detailed analysis of 19 individual free amino acids showed that 18 of them increased over time, whereas serine decreased gradually (Supplementary Fig. S3 available at *JXB* online). These results indicate that the *in vitro* cultured berries maintain active exchange with the culture medium and efficiently use the absorbed nutrients for metabolism. These features of the *in vitro* culture system prompted further testing of a wider range of sucrose concentrations and sugar types to detail the effects of sugar supply on anthocyanin accumulation.

### Sucrose supply enhances anthocyanin accumulation in the absence of exogenous ABA supply

**Fig. 3.**
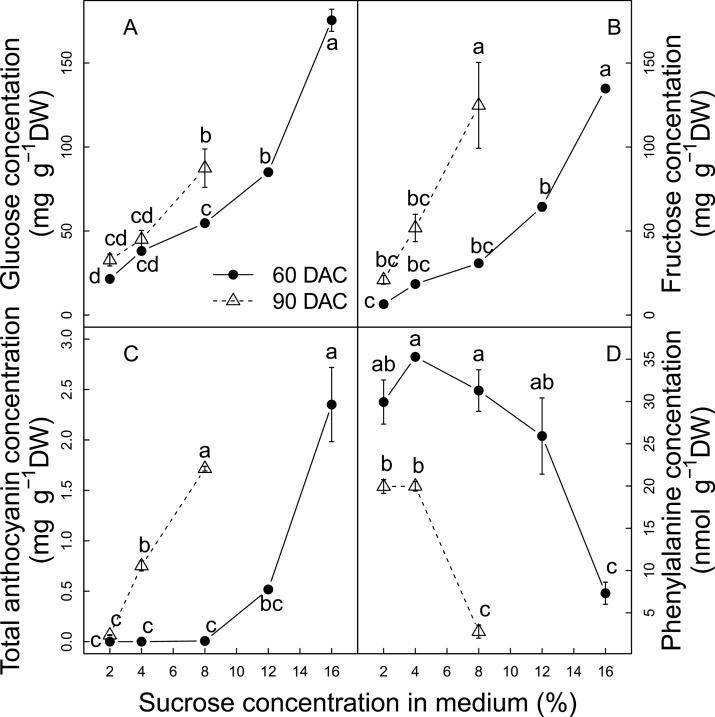
Responses of glucose (A), fructose (B), total anthocyanins (C), and phenylalanine (D) to sucrose supply in the culture medium. Each value corresponds to a mean ±standard error (*n*=3). Different letters indicate significant differences (*P*<0.05) among comparisons of date and medium.

Five sucrose concentrations (2, 4, 8, 12, and 16%) were supplied in the culture media to verify the effect of sugar concentrations on anthocyanin biosynthesis in the detached berries (Fig. 3; Supplementary Fig. S4 available at *JXB* online). Sugar concentration in the berries increased with the sucrose concentration in the culture medium (SCM) and the duration of *in vitro* culture (Fig. 3A, B). At 60 d after starting the culture *in vitro* (DAC, days after culture), glucose and fructose contents in berry were significantly increased when SCM varied from 2% to 16%, with glucose increasing from 21mg g^–1^ DW to 175mg g^–1^ DW and fructose from 6mg g^–1^ DW to 134mg g^–1^ DW. In addition, when berries cultured in the presence of low sucrose concentrations (2, 4, and 8%) were left to grow during an additional 30 d, (sampling at 90 DAC), the berry sugar concentration continued to increase. The berries in 8% sucrose medium reached the same sugar level at 90 DAC as those in 12% sucrose medium at 60 DAC. This indicated the importance of both the sugar concentration and the duration of culture for the final berry sugar concentration. For a given duration of culture, higher SCM resulted in a higher sugar import rate and higher berry sugar concentration. In addition, these results confirmed those from the ^13^C and ^15^N labelling experiments where the active import of sugar from medium to berry was observed.

Anthocyanin levels increased in parallel with the increase of berry sugar concentration as a function of SCM (Fig. 3C). At 60 DAC, only berries in 12% and 16% SCM accumulated a detectable amount of anthocyanins, and these increased significantly with SCM, while those in 2, 4, and 8% were still green (Fig. 3C; Supplementary Fig. S4 available at *JXB* online). At 90 DAC, all berries in 2, 4, and 8% sucrose contained detectable anthocyanin amounts and the contents in 4% and 8% were significantly higher than their counterparts at 60 DAC. These increases in total anthocyanins resulted from the accumulation of peonidin- and malvidin-derived anthocyanins, while petunidin-, delphinidin-, and cyanidin-derived anthocyanins remained in very low quantities and were less responsive to SCM (Supplementary Fig. S5 available at *JXB* online). Since sugar content in a berry primarily depends on the sugar import rate and duration of uptake, the SCM most probably exerts its effects through modifying sugar import. Moreover, the response of anthocyanin to SCM may result not only from the increase in berry sugar concentration, but also from differences in the osmotic potential in the culture media, whose osmotic potentials were not balanced by mannitol. Several authors have reported that higher osmotic potential in the culture medium enhances anthocyanin accumulation in grape cell suspension system (Do and Cormier, 1990, 1991a).

In addition, the results clearly show that exogenous ABA is not needed and that sugar alone can trigger anthocyanin synthesis for green berries cultured *in vitro*, in contrast to the report by Gambetta *et al.* (2010). This discrepancy might result from the shorter *in vitro* culture duration (only 23 d) used in the study of Gambetta *et al.* (2010), and their berries may also have changed colour if they were cultured for a longer period of time (e.g. 60 d or 90 d, as in the present experiment). It would be interesting to check the levels of ABA after sugar addition, in order to clarify whether high sugar triggers *de novo* ABA biosynthesis in grape berries.

The mechanisms underlying the observed positive correlation between sugar concentration and anthocyanin accumulation in grape berry are still unknown. The variation of the concentration of the precursor (phenylalanine) of anthocyanin synthesis was monitored to determine if the sugar effects are mediated by higher levels of anthocyanin precursors. Surprisingly, the phenylalanine concentration was decreased by the increase in SCM (Fig. 3D). At 60 DAC, the phenylalanine concentration decreased from 30 nmol g^–1^ DW under 2% SCM to 7.3 nmol g^–1^ DW under 16% SCM. At 90 DAC, berries had a lower phenylalanine concentration than those at 90 DAC and decreased from 19.9 nmol g^–1^ DW under 2% SCM to 2.8 nmol g^–1^ DW under 8% SCM. This suggests that the sugar effects on anthocyanin are not mediated by increasing the availability of that precursor of anthocyanin synthesis.

### Glucose and fructose are more efficient than sucrose in enhancing anthocyanin accumulation

In *Arabidopsis*, sucrose is more efficient than glucose and fructose in reinforcing anthocyanin accumulation, and the sugar-dependent enhancement of anthocyanin biosynthesis is sucrose specific (Solfanelli *et al.*, 2006). However, it is well known that grape berries predominantly accumulate glucose and fructose, with trace levels of sucrose (Dai *et al.*, 2011), although the accumulated hexoses originate from the sucrose transported in the phloem (Swanson and El-Shishiny, 1958). This high concentration of hexoses may modify the efficiency of different sugar forms in anthocyanin accumulation compared with *Arabidopsis*. Therefore, the effects of sucrose, glucose, and fructose on anthocyanin accumulation were tested by supplying three concentrations (58, 234, and 468mM corresponding to 2, 8, and 16% for sucrose) in the culture media.

**Fig. 4.**
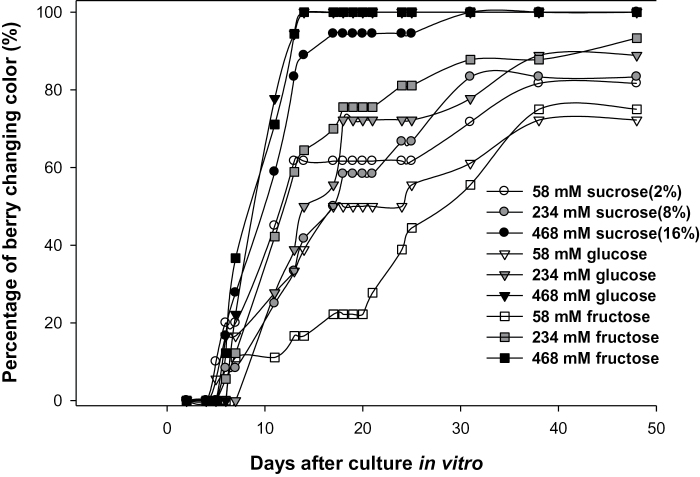
Dynamic colour change of grape berries cultured *in vitro* in response to sugar supply levels and sugar forms.

Berries exposed to high sugar concentrations changed their colour much faster than those cultured in lower sugar media, regardless of sugar types (Fig. 4). Almost all berries cultured in the highest SCM (468mM) changed colour after 14 DAC (100% of berries for glucose and fructose and 90% for sucrose). In contrast, the rate of colour change was slowed down for berries cultured in 58mM and 234mM sugars, with only ~50% of berries changing their colour at 14 DAC. Since the osmotic potential of the culture medium was balanced by mannitol, the observed effects were not due to differences in osmotic potential but indicate a real effect of sugar concentration as observed in *Arabidopsis* (Teng *et al.*, 2005; Solfanelli *et al.*, 2006).

**Fig. 5.**
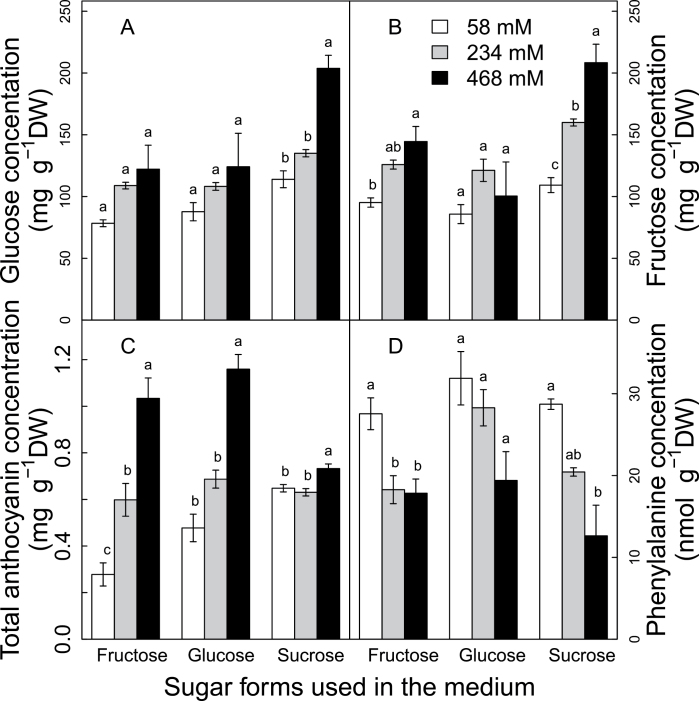
Responses of glucose (A), fructose (B), total anthocyanins (C), and phenylalanine (D) to sugar supply levels and sugar forms in the culture medium. Each value corresponds to a mean ±standard error (*n*=3). Different letters indicate significant differences among sugar concentrations within a given sugar type at *P*<0.05.

In agreement with results from the sucrose concentration experiment, a higher sucrose concentration in the culture medium led to higher hexose concentrations (glucose and fructose) in berries (Fig. 5A, B). Hexose concentrations of berries cultured in glucose medium and the glucose concentration of berries cultured in fructose medium were also increased by increasing hexose concentrations in the medium. Total anthocyanins were increased by rising sugar concentrations in the culture medium for all sugar types, but the extent of increase was sugar type dependent (Fig. 5C). Fructose and glucose were more effective in enhancing anthocyanin accumulation in berries as compared with sucrose. From 58mM to 468mM sugar, the anthocyanin contents of berries in fructose- and glucose-supplemented media increased ~3-fold (from 0.38mg g^–1^ DW to 1.09mg g^–1^ DW), while those in sucrose medium only increased by 14% (from 0.65mg g^–1^ DW to 0.73mg g^–1^ DW). These increased anthocyanin levels were not due to an increase in the precursor concentration (phenylalanine) and, conversely, this amino acid was significantly decreased by increasing sugar concentration in the media (Fig. 5D).

### Anthocyanin is positively correlated with total sugar and negatively correlated with phenylalanine in grape berry

**Fig. 6.**
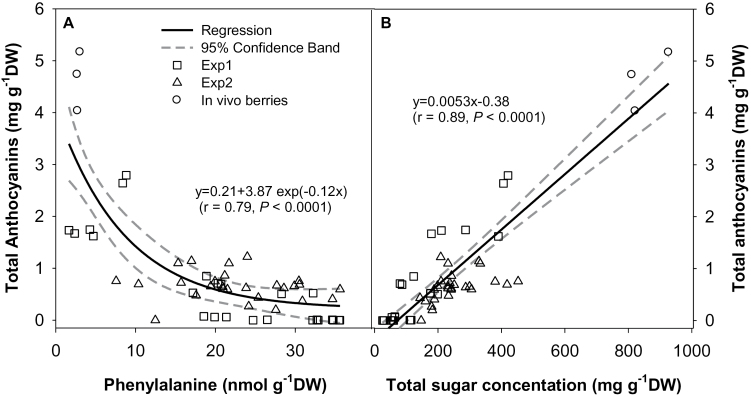
Correlation between total anthocyanins and phenylalanine (A) or total sugar (B) concentrations. Data from experiments on sucrose concentration (experiment 1) and sugar forms (experiment 2) and from berries (*in vivo*) harvested from the parent vines at maturity were pooled.

There was a common non-linear negative correlation between phenylalanine and total anthocyanins, regardless of sugar supply levels or forms tested in the present study (Fig. 6A). Phenylalnine is the starting precursor of the phenylpropanoid pathway that leads to the biosynthesis of anthocyanins. Addition of phenylalanine (from 0 to 4mM) in the culture medium increases anthocyanin production in grape cell suspensions (Krisa *et al.*, 1999; Saigne-Soulard *et al.*, 2006). The negative correlation observed here suggests that a lower anthocyanin accumulation in berries cultured in low sugar media is not due to phenylalanine limitation. It rather points towards a limitation in the biosynthetic enzyme activities that cause a lower rate of phenylalanine transformation to anthocyanins, thereby resulting in higher phenylalanine levels in berries cultured in low sugar media. Furthermore, a common positive linear correlation was observed between total sugar concentration and anthocyanins (Fig. 6B). From this linear function, a threshold value of sugar (71.7mg g^–1^ DW), above which berries are stimulated to accumulate anthocyanins, could be obtained. This type of sugar threshold value seems also to exist for *in vivo* berries, which start to change colour only when the berry sugar concentration is >9–10 °Brix (i.e. 612mg g^–1^ DW) (Keller, 2010).

### Sugar supply alters metabolic channelling between the blue-coloured delphinidin derivates and red-coloured cyanidin derivatives

**Fig. 7.**
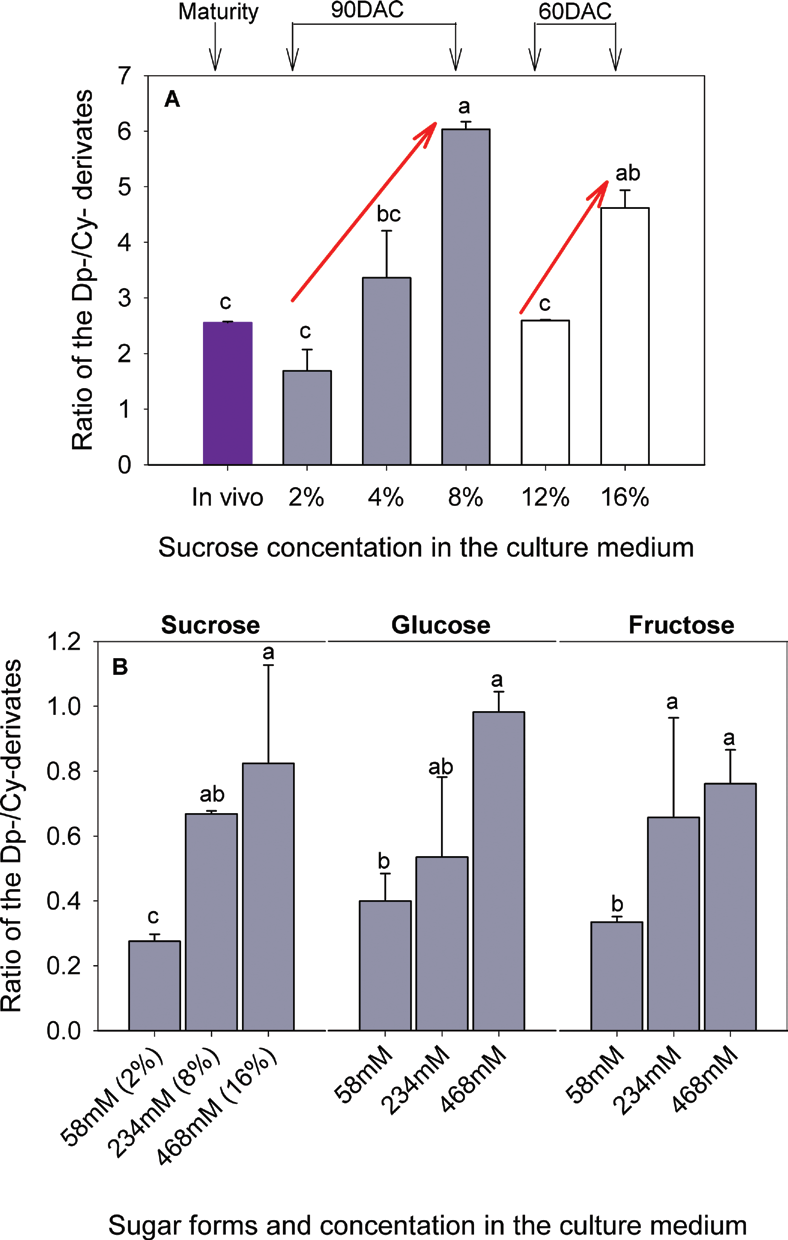
Modification of the ratio between blue-coloured delphinidin-derived (Dp-) and red-coloured cyanidin-derived (Cy-) anthocyanins by sugar levels (A) and sugar forms (B) in the culture medium for berries cultured *in vitro*. Results in the berries (*in vivo*) harvested from the parent vines at maturity were supplied as a control. Each value corresponds to a mean ±standard error (*n*=3). Different letters indicate significant differences among sugar concentrations within a given sugar type at *P*<0.05.

Five classes of anthocyanins (cyanidin-, peonidin-, delphinidin-, petunidin-, and malvidin-derived anthocyanins) are usually present in berry skin of red wine grape cultivars (Supplementary Fig. S5 available at *JXB* online). Cyanidin- and petunidin-derived anthocyanins confer red colour, while delphinidin-, malvidin-, and peonidin-derived anthocyanins confer blue colours. In addition to the total anthocyanin content, sugar supply also affected the composition of anthocyanins (Fig. 7). At 60 DAC, the ratio of delphinidin- to cyanidin- derived anthocyanins increased from 2.6 to 4.6 when the sucrose concentration in the media increased from 12% to 16%. At 90 DAC, a 3.6-fold increase was observed when the sucrose concentration increased from 2% to 8%. Similar increases in the delphinidin/cyanidin derivative ratio were evident after addition of glucose or fructose. This ratio is known to be enhanced by water stress (Castellarin *et al.*, 2007a, b) and to vary with grape genotypes (Castellarin *et al.*, 2006; Castellarin and Di Gaspero, 2007) and berry developmental stage (Falginella *et al.*, 2012). The variation in the delphinidin/cyanidin derivate ratio is tightly related to the ratio of expression of flavonoid 3′,5′-hydroxylase (F3′5′H) to flavonoid 3′-hydroxylase (F3′H), which channel, respectively, metabolic fluxes to the delphinidin- and cyanidin-derived anthocyanins (Castellarin *et al.*, 2006). The microarray results (see detailed discussion below) showed that the expression ratio F3′5′H/F3′H was higher for berries grown under a higher glucose concentration (Supplementary Fig. S6 available at *JXB* online), confirming that the relative expression levels of F3′5′H and F3′H play a key role in determining anthocyanin composition.

### Sugar supply triggers massive transcriptome reprogramming

Microarray reproducibility and reliability were first verified through a heatmap of sample distance and hierarchical clustering (Supplementary Fig. S7 available at *JXB* online). The samples were clearly separated into two groups corresponding to the two media with different glucose concentrations. Within each group, the three biological replicates for a given treatment were closely related, indicating good reproducibility and reliable hybridization.

**Fig. 8.**
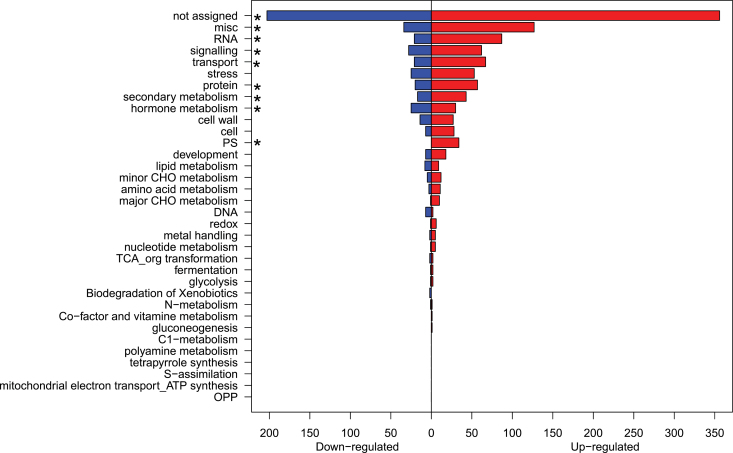
Distribution of the Mapman functional categories of genes significantly down- or up-regulated in berries cultured *in vitro* in high glucose (468mM) compared with low glucose (58mM) in the culture medium. A total of 1515 significantly differentially expressed genes were identified by an adjusted *P*<0.05 after false discovery rate correction. Three biological replicates were used. Asterisks indicate that the functional categories of the MapMan ontology are significantly enriched in the significantly differentially expressed genes at *P*<0.05 after Bonferroni correction. (This figure is available in colour at *JXB* online.)

A total of 1515 transcripts were identified as significantly differentially expressed between berries cultured in medium with a high concentration of glucose (468mM) in comparison with those cultured in a low concentration of glucose (58mM). Among them, 1058 transcripts were up-regulated by a high glucose concentration in the culture medium and 457 were down-regulated (Fig. 8). Functional ontology enrichment analysis showed that the significantly differentially expressed genes were involved in several major functional categories, including transcription factors, signalling, transport, stress response, protein (post-translational modification, degradation), secondary metabolism, hormone metabolism (ABA, auxin, brassinosteroid, cytokinin, ethylene, jasmonate, and salicylic acid), and cell wall metabolism, which account for 80% of significant transcripts with an annotated functional category (Supplementary Table S1 available at *JXB* online). These results are consistent with those reported for a genome-wide transcriptome analysis of field-sampled berries, when comparing ripening (post-véraison) versus green (pre-véraison) fruits (Deluc *et al.* 2007; Pilati *et al.*, 2007). For the sake of simplicity, the present study focused on a discussion of the genes related to sugar signalling and anthocyanin biosynthesis.

Some regulatory genes involved in sugar signalling were modified by the glucose concentration in the culture medium. A putative hexokinase was up-regulated 2.5-fold (*P*<0.001) by higher glucose in the culture medium; WRKY transcriptional factors were down-regulated ~2.5-fold (*P* <0.01); and Snf1-like protein kinase was down-regulated ~2.3-fold (*P*<0.05). The results of hexokinase expression variations were in agreement with the findings of Gambetta *et al.* (2010) who showed that hexokinase was induced by the ripening process, coinciding with sugar accumulation in field-grown berries. In addition, Vitrac *et al.* (2000) have postulated that hexokinase is involved in sugar signalling transduction leading to anthocyanin accumulation. All annotated transcripts involved in ABA signalling were down-regulated by a high glucose medium, including homeobox (HB) transcription factors (~3-fold) and *AP2* (~2.4-fold) in this study. However, the HB transcription factors have been shown to be induced more in berries cultured in a medium of 10% sucrose+10/200 μM ABA than in those cultured in 2% sucrose or 10% sucrose (Gambetta *et al.*, 2010). This difference between the two studies most probably arises from the fact that Gambetta *et al.* (2010) used ABA in the media, and ABA induced ABA-related genes such as HB transcription factors and APs, while sugar treatments carried out in the present study are not inducing ABA-related genes. In addition, the vacuolar invertase *GIN2* was down-regulated ~4-fold (*P*<0.05) by a high glucose concentration in the culture medium. Calcium signalling genes were mostly up-regulated by higher glucose in the medium, for example a homologue gene encoding calmodulin-related protein was increased 2-fold (*P*<0.01). This calmodulin-related protein may play a role in the calmodulin-dependent signal transduction pathways that mediate sugar induction of anthocyanin synthesis (Vitrac *et al.*, 2000).

**Fig. 9.**
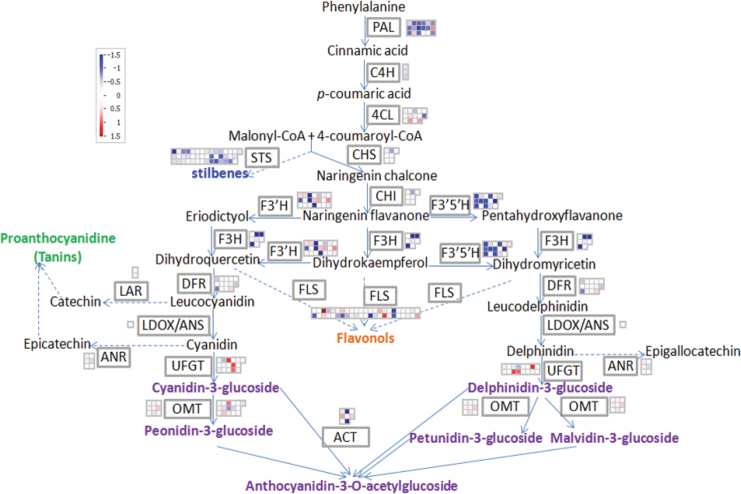
Effect of glucose concentration (468/58mM) in the culture medium on the expression of structural genes involved in anthocyanin biosynthesis in grape berries cultured *in vitro*. Expression data from the microarray were visualized using Mapman software. Genes involved in each metabolic step are located beside their encoded enzyme names. Relative expression levels calculated as log2 ratios (468mM/58mM) are represented as false colour with red for up-regulated genes and blue for down-regulated genes as illustrated in the colour key. Abbreviations: *PAL*, phenylalanine ammonia-lyase; *C4H*, cinnamate-4-hydroxylase; *4CL*, 4-coumarate-CoA ligase; *CHS*, chalcone synthase; *STS*, stilbene synthase; *CHI*, chalcone isomerase; *F3*′*H*, flavonoid 3′-hydroxylase; *F3H*, flavonone 3-hydroxylase; *F3*′*5*′*H*, flavonoid 3′5′-hydroxylase; *DFR*, dihydroflavanol 4-reductase; *FLS*, flavonol synthase; *LDOX/ANX*, leucoanthocyanidin dioxygenase/anthocyanidin synthase; *UFGT*, UDP-glucose:anthocyanidin 3-*O*-glucosyltransferase; *OMT*, *o*-methyltransferase; *ACT*, anthocyanin acyltransferase; *LAR*, leucoanthocyanidin reductase; *ANR*, anthocyanidin reductase.

In addition to the alterations of regulatory gene expression, structural genes involved in the flavonoid biosynthetic pathway were also induced or repressed by a high glucose concentration (Fig. 9). The down-regulated genes included a putative *PAL* gene (2.6-fold, *P*<0.01), a putative *F3H* (~4-fold, *P*<0.01), three homologues of *F3*′*H* (~3.8-fold, *P*<0.01), a putative *F3*′*5*′*H* (~2.4-fold, *P*<0.01), and a putative *ACT* (4.6-fold, *P*<0.001). In contrast, putative *UFGT* (UDP-glucose:anthocyanidin 3-*O*-glucosyltransferase) genes were up-regulated ~2-fold (*P*<0.05), while the remaining structural genes involved in the anthocyanin pahway (*C4H*, *4CL*, *CHS*, *CHI*, *DFR*, *LDOX/ANS*, and *OMT*) were not significantly modified.

Apart from the branches leading to anthocyanin synthesis, other metabolic branches were also responsive to the glucose concentration of the medium. *STS* involved in the stilbene metabolic branch was down-regulated ~2.6-fold (*P*<0.05), while *FLS* in the flavonol metabolic branch was up-regulated ~3-fold (*P*<0.01) by high glucose. These results clearly indicate that the flavonoid metabolic pathway was modified by glucose supply and may be responsible for the observed enhancement of anthocyanin content.

When comparing the present results with those available in the literature, both consensus and discrepancies for gene expression responses to sugar treatment were found. In *Arabidopsis* seedlings, both glucose and sucrose induce anthocyanin production, but sucrose was more effective than glucose in triggering gene expression in the anthocyanin biosynthesis pathway (Teng *et al.*, 2005; Solfanelli *et al.*, 2006). In addition, the extent of up-regulation by sucrose varies for genes located at different positions of the pathway: downstream genes *DFR*, *LDOX/ANS*, and *UFGT* were more induced than upstream genes *PAL*, *C4H*, *4CL*, *CHS*, *CHI*, *F3H*, and *F3*′*H* (Solfanelli *et al.*, 2006). This effect of sugar was not mediated via osmotic potential variations but was really sugar specific (Solfanelli *et al.*, 2006). In radish detached hypocotyls, *PAL*, *CHS*, *CHI*, *F3H*, *DFR*, and *LDOX/ANS* transcript levels were significantly increased by the presence of 175mM sucrose, in agreement with enhanced anthocyanin content (Hara *et al.*, 2003, 2004). These authors did not examine the response of other structural genes or the effects of glucose or fructose. In grape berry slices, sugars (glucose 100mM, fructose 100mM, and sucrose 150mM) were able to induce transient expression of *F3H* from 2h to 4h (Zheng *et al.*, 2009). Concerning other genes, limited information is available in grape berry, especially concerning the hexose effect. During berry development, anthocyanin accumulation is paralleled by a coordinated up-regulation of most genes (except *UFGT*) involved in the anthocyanin synthesis pathway (Boss *et al.*, 1996). However, *UFGT* is not expressed in concert with the other genes and functions as a major control point in anthocyanin synthesis (Boss *et al.*, 1996). Moreover, *UFGT* expression is tightly correlated with tissue anthocyanin content in grape berry (Castellarin and Di Gaspero, 2007; Zheng *et al.*, 2013). Therefore, the up-regulation of *UFGT* observed in the present experiments, together with that of regulatory genes, certainly plays a role in enhancing the anthocyanin level in berries cultured in high glucose medium.

### Conclusions

An experimental system allowing the long-term *in vitro* culture of grape berries (up to 90 d without contamination) was designed and validated. This system was based on the combination of production of fruiting-cuttings and *in vitro* organ culture techniques. The berries cultured with this system were able to absorb carbon and nitrogen compounds from culture media and to use them for metabolism, as revealed by ^13^C and ^15^N labelling experiments. The potential of this *in vitro* culture system was further illustrated by studying the effects of sugar concentration and sugar types on anthocyanin accumulation. The results showed that a sucrose concentration >2% induces anthocyanin synthesis without the need for an exogenous ABA supply. Enhanced anthocyanin accumulation could be induced by glucose, fructose, and sucrose, with glucose and fructose being more effective than sucrose. The increase in anthocyanin accumulation under higher sugar supply was not due to limitation of its precursor, since anthocyanins were negatively correlated with their precursor phenylalanine. Instead, it probably resulted from a modification of the expression of regulatory and structural genes (especially *UGFT*) of the flavonoid biosynthesis pathway by high sugar concentrations, as highlighted by genome-wide transcriptome analysis. In addition to the sugar-induced alternation in total anthocyanins, the ratio between blue-coloured delphinidin derivates and red-coloured cyanidin derivates was also increased by higher sugar supply. This increase is related to the expression ratio of *F3*′*5*′*H* to *F3*′*H* genes, which drive, respectively, metabolic fluxes to the delphinidin- and cyanidin-derived anthocyanins. In the future, this *in vitro* culture system can be used to study other nutritive, environmental, and hormonal factors that are known to modify anthocyanin accumulation, such as nitrogen supply (Do and Cormier, 1991b; Hilbert *et al.*, 2003), phosphate concentration (Yamakawa *et al.*, 1983; Yin *et al.*, 2012), ABA (Gambetta *et al.*, 2010; Deis *et al.*, 2011), light (Zheng *et al.*, 2013), and temperature (Boss and Davies, 2009).

## Supplementary data

Supplementary data are available at *JXB* online.

Figure S1. Home-made floater device used in the liquid *in vitro* culture system for grape berries.

Figure S2. Pictures of the liquid *in vitro* culture system for tracing the uptake of ^13^C and ^15^N.

Figure S3. Profiles of 19 individual free amino acids as a function of culture duration in grape berries cultured *in vitro*.

Figure S4. Colour changing of berries cultured *in vitro* with media containing different sucrose concentrations.

Figure S5. Response of anthocyanin composition to sugar concentration and sugar forms in the culture media for grape berries cultured *in vitro.*

Figure S6. Relative transcript abundance of F3′5′H to F3′H in berries cultured *in vitro* under two glucose concentrations.

Figure S7. Heatmap representation of the correlation between arrays.

Table S1. List of genes up- or down-regulated by high glucose in the culture medium for grape berries cultured *in vitro*.

Supplementary Data

## Abbreviations:

ABA: abscisic acid
AP2: Apetala 2 transcriptional factor family
DAC: days after culture
DW: dry weight
EDTA: ethylenediaminetetraacetic acid
F3H: flavonone 3-hydroxylase
F3′H: flavonoid 3′-hydroxylase
F3′5′H: flavonoid 3′5′-hydroxylase
SCM: sucrose concentation in the culture medium
UFGT: UDP-glucose:anthocyanidin 3-*O*-glucosyltransferase.
