# Supplementary material for: Long-term in vitro culture of grape berries and its application to assess the effects of sugar supply on anthocyanin accumulation
Source: J Exp Bot. 2014 Jan 29;65(16):4665–77. doi: 10.1093/jxb/ert489 (PMC4115254; doi:10.1093/jxb/ert489)
Supplement: Supplementary Data [file supp_65_16_4665__index.html]

Long-term in vitro culture of grape berries and its application to assess the effects of sugar supply on anthocyanin accumulation — Long-term in vitro culture of grape berries and its application to assess the effects of sugar supply on anthocyanin accumulation — Supplementary Data 

# Long-term *in vitro* culture of grape berries and its application to assess the effects of sugar supply on anthocyanin accumulation

## Supplementary Data

Data files

**Files in this Data Supplement:**

- Supplementary Data - Supplementary Data
- Supplementary Data - Supplementary Data
