# Supplementary material for: Long-term in vitro culture of grape berries and its application to assess the effects of sugar supply on anthocyanin accumulation
Source: J Exp Bot. 2014 Jan 29;65(16):4665–77. doi: 10.1093/jxb/ert489 (PMC4115254; doi:10.1093/jxb/ert489)
Supplement: Supplementary Data [file supp_ert489_jexbot108639_file002.pdf]

Long-term *in vitro* culture of grape berries and its application to assess the effects of sugar supply on anthocyanin accumulation

Zhan Wu Dai[1]*, Messaoud Meddar[1], Christel Renaud[1], Isabelle Merlin[1], Ghislaine Hilbert[1], Serge Delrot[2], Eric Gomès[2]

[1] INRA, ISVV, UMR 1287 EGFV, 33882 Villenave d'Ornon, France

[2] Univ. de Bordeaux, ISVV, UMR 1287 EGFV, 33882 Villenave d'Ornon, France

*corresponding author: zhanwu.dai@bordeaux.inra.fr, tel: 33 (0)5 57 57 59 22, fax: 33 (0)5 57 57 59 03

Journal of Experimental Botany, 2013

Supplementary Table S1. Osmotic pressures of different culture media used in Exp1 and Exp2. Osmotic pressure is calculated with the van 't Hoff equation (online calculator: http://hyperphysics.phy-astr.gsu.edu/hbase/kinetic/ospcal.html).

Exp	Medium	Osmotic pressure (MPa)
1	2%	0.38
	4%	0.52
	8%	0.82
	12%	1.10
	16%	1.40
2	2%	1.40
	8%	1.40
	16%	1.40

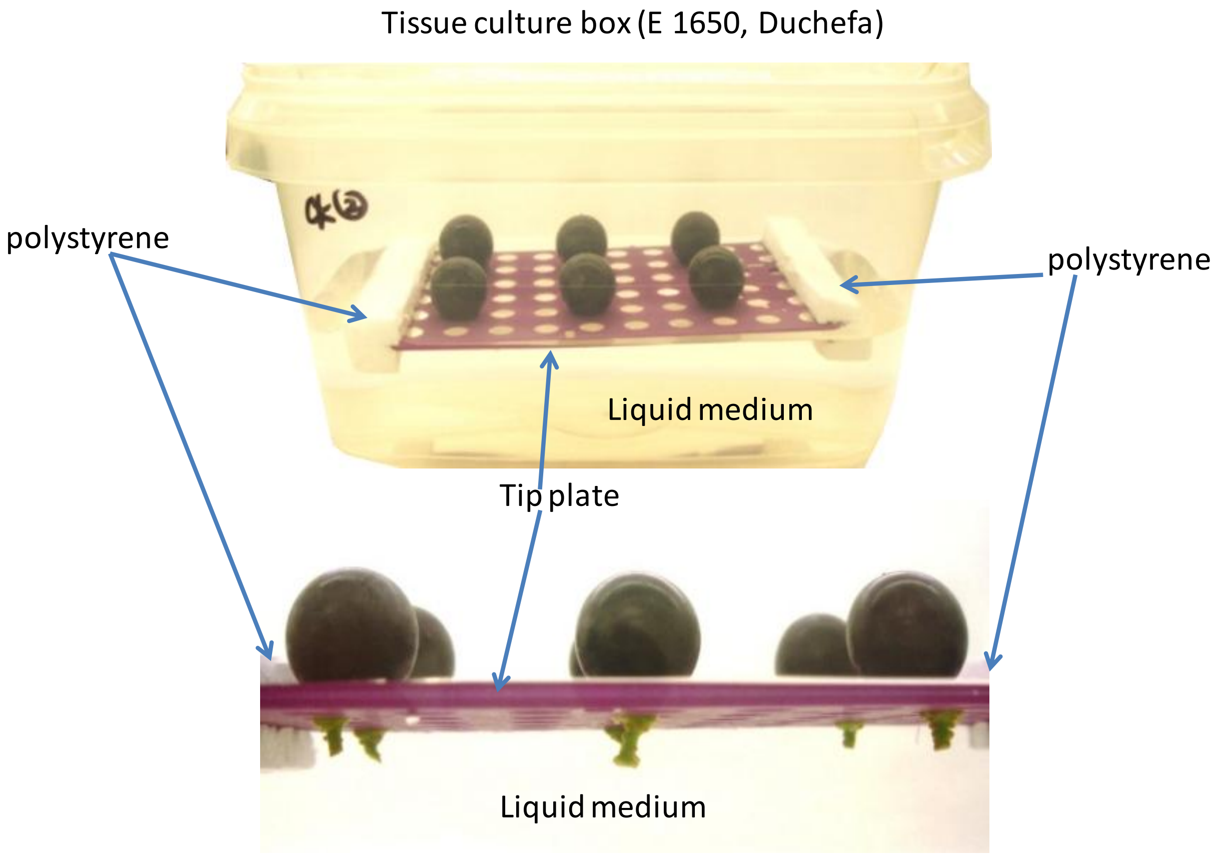

Supplementary Figure S1. Illustration of the homemade floater used in the liquid *in vitro* culture system for grape berries.

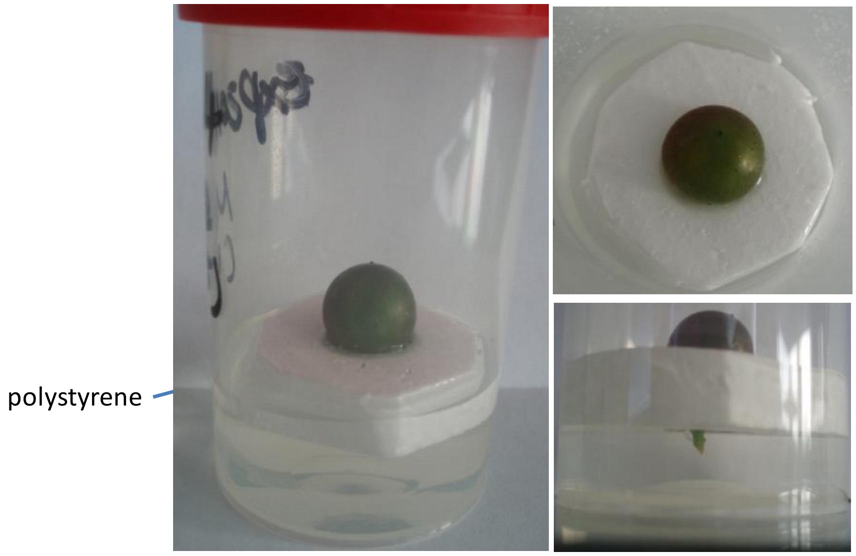

Supplementary Figure S2. Illustration of the liquid *in vitro* culture system for tracing the uptake of ^{13}C and ^{15}N.

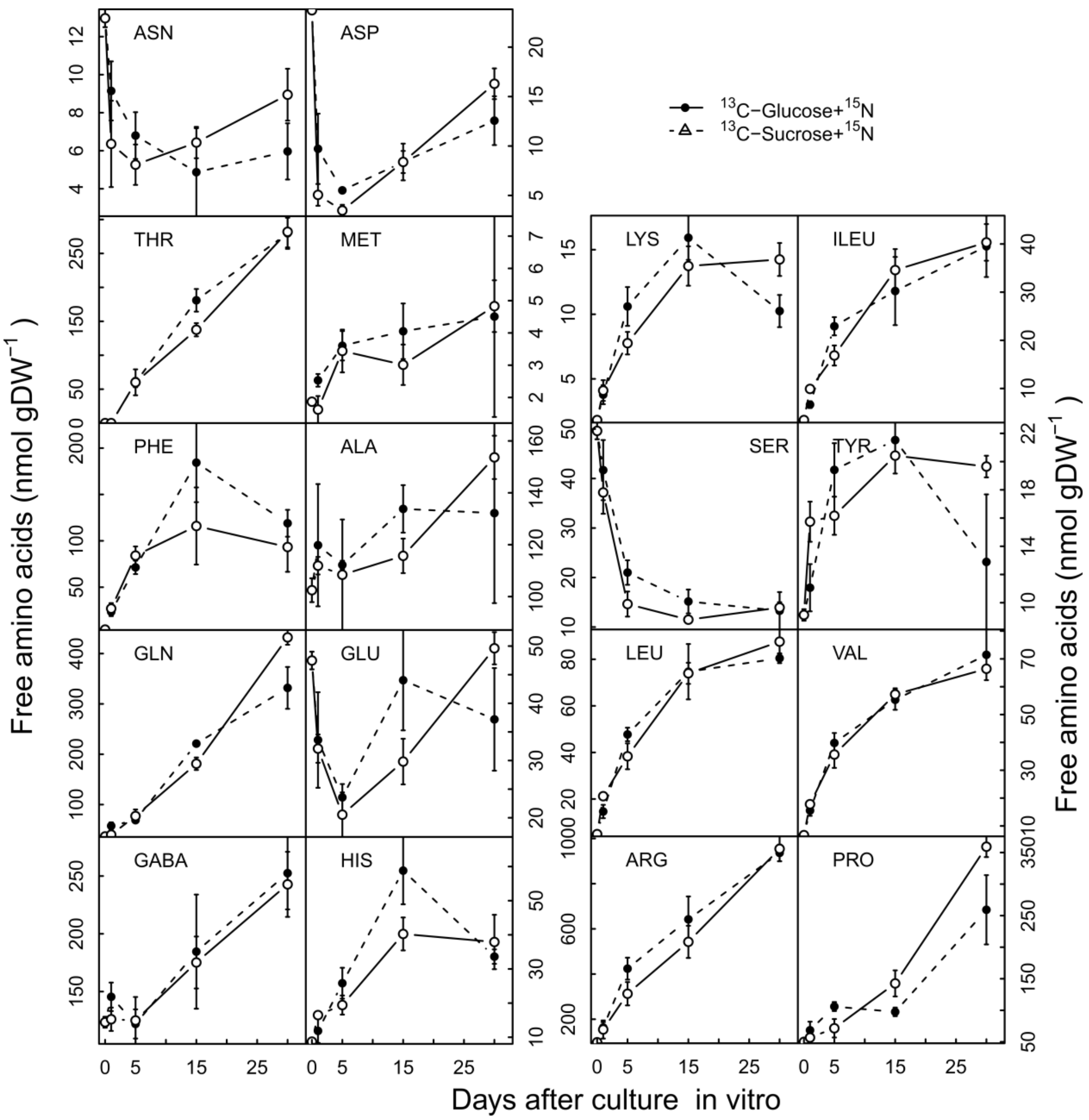

Supplementary Figure S3. Profiles of 19 individual free amino acids as a function of culture duration in grape berries cultured *in vitro*. The culture media contain ^{13}C and ^{15}N labeled carbon and nitrogen resources. Each value corresponds to a mean ± standard error (n = 3).

Treatment	0 DAC	60DAC	90 DAC
2% Sucrose	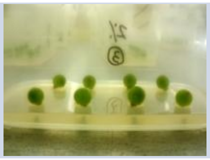	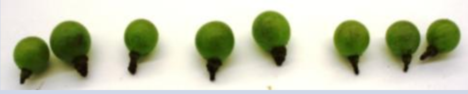	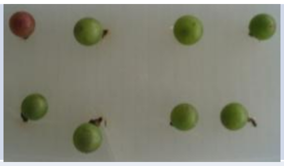
4% Sucrose	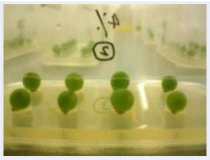	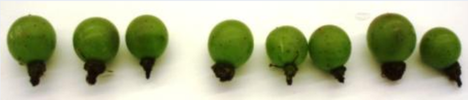	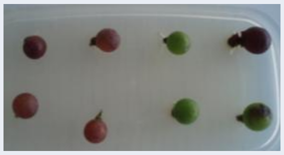
8% Sucrose	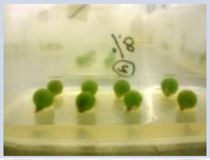	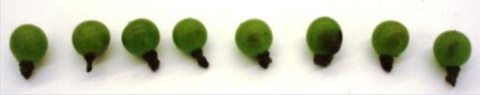	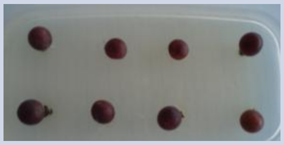
12% Sucrose	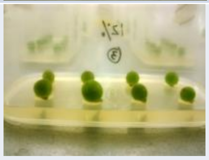	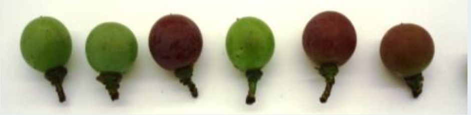	
16% Sucrose	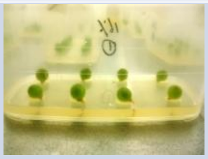	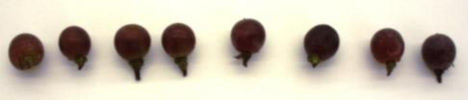	

Supplementary Figure S4. Color changing of berries cultured *in vitro* with media containing different sucrose concentrations. DAC=days after culture *in vitro.*

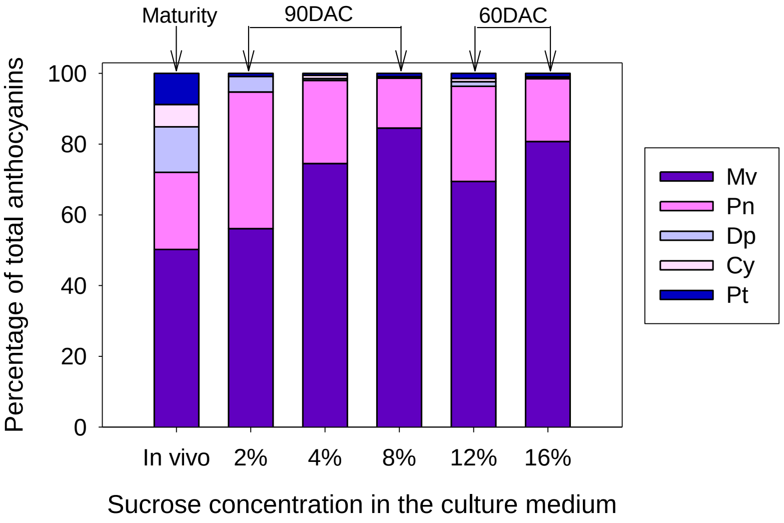

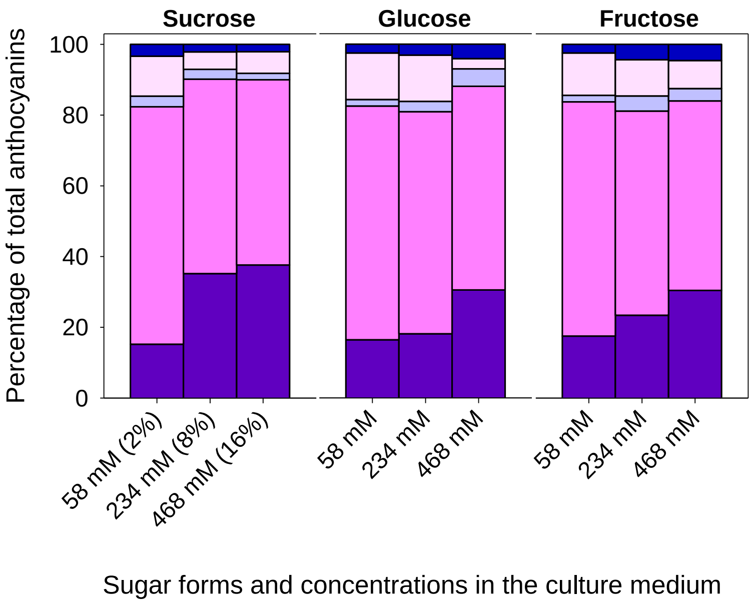

Supplementary Figure S5. Response of anthocyanin composition to sugar concentration and sugar forms in the culture media for grape berries cultured *in vitro.* Each value corresponds to the mean of 3 biological replicates. Mv = malvindin-derivates; Pn = peonidin-derivates; Dp = delphinidin-derivates; Cy = cyanidin-derivates; Pt = petunidin-derivates.

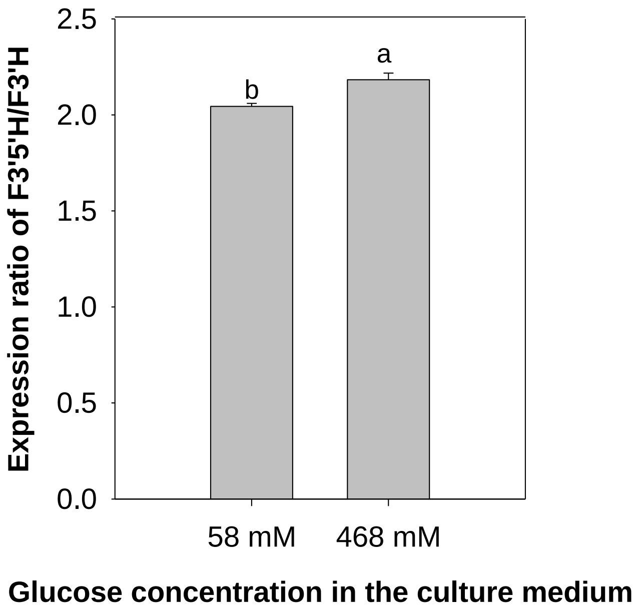

Supplementary Figure S6. Relative transcript abundance of F3'5'H to F3'H in berries cultured *in vitro* under two glucose concentrations. Each value corresponds to a mean ± standard error (n = 3). Different letters indicate significant differences after t test at $P < 0.05$.

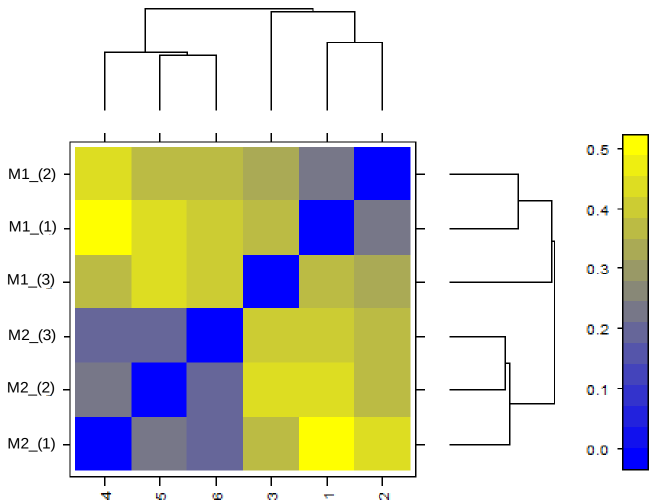

Supplementary Figure S7. Heatmap representation of the distance between arrays. The dendrogram on the top and right showed the arrays clusters. The color scale is chosen to cover the range of distances encountered in the dataset. Berries cultured in two media with different glucose concentrations were named as M1 for 54 mM and M2 for 468 mM with their biological replicate number indicated inside the parentheses.
